# Early-stage clinical outcomes and rotational stability of TECNIS toric intraocular lens implantation in cataract cases with long axial length

**DOI:** 10.1186/s12886-020-01465-2

**Published:** 2020-05-25

**Authors:** Suhong He, Xiang Chen, Xingdi Wu, Yajuan Ma, Xuewen Yu, Wen Xu

**Affiliations:** 1 Eye Center of the Second Affiliated Hospital, Zhejiang University School of Medicine, No. 88 Jiefang Road, Hangzhou, China; 2 Suichang Hospital of Traditional Chinese Medicine, Suichang, China

**Keywords:** Astigmatism, Cataract, Toric IOL rotation, Area of capsulorhexis

## Abstract

**Background:**

A major focus of toric intraocular lens (IOL) implantation is the rotational stability, especially in the patients with long axial length (AL). In this study, we aimed to evaluate the clinical outcomes after implantation of TECNIS toric IOL in eyes with long AL and identify factors influencing their early-stage stability with preoperative corneal astigmatism.

**Methods:**

The study population consisted of 64 eyes from 52 cataract patients, and these patients had preoperative corneal astigmatism between 1.0 and 3.7 diopters (D) and underwent phacoemulsification and TECNIS toric IOL implantation. Ophthalmic biological measurements were carried out preoperatively, including AL, anterior chamber depth (ACD), lens thickness (LT), vitreous length (VL), anterior chamber volume (ACV), sulcus-to-sulcus (STS) and keratometric value (K). Clinical examinations, including visual acuity, manifest refraction, keratometry, digital anterior segment photographs with pupillary dilation, were performed at 1 and 3 months after surgery.

**Results:**

The mean best corrected distance visual acuity (BCDVA) was improved from 0.93 ± 0.35 logarithms of the minimal angle of resolution (logMAR) preoperatively to 0.07 ± 0.10 logMAR postoperatively at 3 months after surgery. The mean residual astigmatism (RAS) was 0.91 ± 0.74D at 3 months, which was significantly decreased compared with the preoperative corneal astigmatism of 1.71 ± 0.55 D. The mean absolute rotation of TECNIS toric IOL at 1 and 3 months was 7.42 ± 11.32 degree (°) (0–79°) and 7.48 ± 11.19°(0–79°), respectively. The mean area of capsulorhexis and the overlapped area between capsulorhexis and IOL optic intraoperatively was 21.04 ± 3.30 mm^2^ and 7.40 ± 2.87 mm^2^.A positive correlation was found between IOL rotation and the area of capsulorhexis (*p* = 0.017) at 3 months after surgery. No correlation was found between IOL rotation and AL (*p* = 0.876), ACD (*p* = 0.387), LT (*p* = 0.523), VL (*p* = 0.546), ACV (*p* = 0.480), STS (*p* = 0.884), K1 (*p* = 0.429), K2 (*p* = 0.644), average of K1 and K2 (*p* = 0.520), intraoperative IOL axial direction (*p* = 0.396), preoperative corneal astigmatism (*p* = 0.269) or the overlapped area between capsulorhexis and IOL optic intraoperatively (*p* = 0.131) .

**Conclusions:**

The large CCC was a risk factor for toric IOL rotation. An appropriately smaller sized CCC was conducive to increase the rotational stability of TECNIS toric IOL implantation in cataract cases with long AL.

## Background

With the development of phacoemulsification in cataract surgery and design of intraocular lens (IOL), better quality of vision has been achieved postoperatively. Patients with long axial length (AL) who have undergone cataract surgery and had IOL implantation may be willing to decrease myopia and residual astigmatism (RAS). Therefore, the management of pre-existing astigmatism has become more clinically important [1]. There are several surgical options for correcting preoperative astigmatism, such as femtosecond laser-assisted astigmatic keratotomy [2], limbal or corneal relaxing incisions [3]_,_laser-assisted in situ keratomileusis (LASIK) [4], and implantation of toric IOLs. Implantation of toric IOL has its superiority of accuracy, predictability and economical efficiency compared with other options. A major problem of toric IOL implantation is the rotational stability. Patients with longer AL accordingly tend to have bigger capsular bags than those with emmetropia or hypermetropia [5, 6]. It indicates that larger capsular bags may reduce the equatorial friction for a given IOL, and thinner IOL may increase the gap between the optical component and the posterior capsule [7], therefore leading to decreased IOL stability. However, some scholars did not find the correlation between the IOL rotation and AL in myopic eyes [8]. Our previous research has discovered the correlation between toric IOL rotation and LT, AL and area of capsulorhexis in eyes of various axial lengths and a positive correlation between AL and toric IOL rotation [9]. The controversy about the possible correlation between the AL and IOL rotation remains furious. In the present study, we focused the toric IOL rotation of cataract patients with long AL eye and aimed to assess the correlation between boimetric measurements and rotational stability of TECNIS toric IOL implantation in cataracts with long AL. Moreover, we attempted to identify the potential factors influencing the rotational stability at early stage after surgery.

## Methods

### Study subjects

A total of 64 eyes that underwent phacoemulsification and implantation of TECNIS toric IOL (AMO Groningen BV, 9728 NX Groningen, the Netherlands) were enrolled in this retrospective non-comparative clinical study. AMO TECNIS toric IOL was a single-piece hydrophobic acrylic aspheric lens with a 6-mm optic diameter and an overall diameter of 13 mm. The IOL had a modified C-loop haptic configuration. The diameter for the outermost and innermost marking holes was 6.1 mm and 4.6 mm, respectively. All enrolled patients underwent cataract surgery from May, 2015 to October, 2018 at the Eye Center, the Second Affiliated Hospital of Zhejiang University. Informed consent was provided from every participant preoperatively. Before surgery, all patients with cataract had a regular total corneal astigmatism (TCA) of ≥1.00 D and an AL ≥25.0 mm. When the difference of the astigmatism meridian of the anterior and posterior surface was within ±10 degrees and astigmatism difference was within ±0.75 D and quality safety (QS) was OK, the total astigmatism shown on Pentacam was used to calculate before surgery. Exclusion criteria included patients with following eye conditions, such as irregular corneal astigmatism, a history of intraocular surgery, pterygium, glaucoma, retinal detachment, uveitis, macular degeneration or retinopathy, lens subluxation, posterior capsule opacification, and abnormal lens morphology. The refractive target of sphere was low myopia or emmetropia.

### Preoperative examinations

Long AL was defined as an AL of 25 mm or greater. Preoperatively, patients had a routine examination for uncorrected distance visual acuity (UCDVA), best corrected distance visual acuity (BCDVA), intraocular pressure (IOP), manifest refraction, slit-lamp examination with pupillary dilation, biometric measurement and toric IOL calculations. The biometric measurement included AL (IOLMaster 500, Carl Zeiss, Advanced Technology V.5.5, Germany), TCA (Pentacam; Oculus Optikgeräte GmbH, Wetzlar, Germany), keratometry (K) and anterior chamber volume (ACV) (Pentacam; Oculus Optikgeräte GmbH, Wetzlar, Germany), anterior chamber depth (ACD) (Pentacam; Oculus Optikgeräte GmbH, Wetzlar, Germany) and sulcus-to-sulcus (STS) (Anterior Segment VisanteTM OCT, Carl Zeiss, Germany). Immersion A-scan biometry (Quantel Medical CineScan AVISO, France) was used to determine the lens thickness (LT) and vitreous length (VL). The spherical power of IOL was examined using SRK-T formula, and the cylinder power and alignment axis were calculated using an online calculator (https://www.TecnisToricCalc.com). Surgically induced astigmatism (SIA) was entered as 0.3 D with a 2.0-mm clear limbal incision, and TCA was taken into account for calculation of the IOL’s cylindrical power. The steep meridian of the corneal was used to determine the incision position.

### Surgical technique

Each patient underwent the same technique by an experienced surgeon (Dr. Wen Xu). With the patients sitting upright on a slit-lamp microscope, the 0 and 180 degree positions of the corneal limbus were precisely marked with a fine syringe needle and highlighted with skin marker (Medplus Inc.) before pupil was dilated by a same skillful assistant. After topical or general anesthesia, toric IOL alignment axis was marked using a Mendez ring and highlighted for recognition under the operating microscope. Generally toric IOL alignment axis was marked on the steep meridian of cornea. A 2.0-mm clear limbal incision was made on the steep meridian with central continuous curvilinear capsulorhexis (CCC) followed by phacoemulsification and cortical aspiration. The toric IOL was inserted into the capsular bag using an injector (DK7786 with One Series Ultra Cartridge Implantation System) and rotated into a position approximately 5° to 10° counter-clock wise from the planned axis. After all the viscoelastic substances were removed from behind and in front of the IOL, the axis was aligned in a clockwise direction to the intended placement. The incisions were hydrated, and the position of the IOL was properly oriented prior to the end of surgery. No sutures were used to close the wound. For analysis of Toric IOL axis, the area of capsulorhexis and the overlapped area between capsulorhexis and IOL optic, the procedures of all surgeries were recorded. The postoperative treatment consisted of prednisolone acetate eye drops (Allergan Pharmaceutical Ireland, Westport, Ireland) and levofloxacin (Cravit, Santen Pharmaceutical) four times a day for 7 days, as well as pranprofen (Pranopulin, Senju Pharmaceutical, Osaka, Japan) and sodium hyaluronate eye drops (URSAPHARM Arzneimittel GmbH, Germany) four times a day for 1 month.

### Postoperative examinations

Postoperatively, patients received examinations at 1 week, 1 month and 3 months. Postoperative 1- and 3-month follow-ups included slit-lamp examination, intraocular pressure, BCDVA, manifest refraction and digital anterior segment photography. Pupil was adequately dilated to visualize the toric axis marks and the edge of capsulorhexis with a mixture of phenylephrine and 0.5% tropicamide (Mydrin-P; Santen Pharmaceutical). The patients seated before slit-lamp microscope (TOPCON SL-D701, Japan) with an upright position, and digital anterior segment photographs (Topcon, Tokyo, Japan) were acquired and recorded. A conjunctive blood vessel or pigment was selected as a reference meridian to eliminate the influence of head tilt or eye rotation. The difference of IOL axial direction between intraoperative actual axis and postoperative alignment, the area of capsulorhexis and the overlapped area between capsulorhexis and IOL optic were calculated using the ruler tool of Rhinoceros 5.0 (Robert McNeel & Assoc, America) for three times. The mean value was selected for statistical analysis.

### Statistical analysis

All statistical analyses were performed using SPSS Statistics 17.0 (SPSS, Chicago, Illinois, USA). Continuous variables were expressed as the mean and standard deviation (SD). The correlations between continuous variables were assessed using Pearson’s correlation analysis. Multiple linear regression analysis was then performed to assess the independent effects of the various factors that might be associated with toric IOL rotation. A probability value of less than 0.05 was considered statistically significant.

## Results

### Preoperative characteristics

A total of 64 eyes from 52 patients, including 35 right and 29 left eyes, were enrolled in the present study. The mean age of patients at the time of surgery was 59.17 ± 17.14 years, ranging from 17 to 83 years. The mean preoperative BCDVA was 0.93 ± 0.35 logMAR. Table 1 shows the statistical characteristics of patients before the cataract surgery. Table 2 lists the TECNIS toric IOL models.

**Table 1:** Patient characteristics before the cataract surgery

Parameters	Value
Age (years)	59.17 ± 17.14 (17–83)
Gender (male/female)	22/30
Operate eye (right/left)	35/29
BCDVA (LogMAR)	0.93 ± 0.35 (1.7–0.2)
AL (mm)	27.15 ± 1.48 (25.0–31.38)
ACD (mm)	2.85 ± 0.27 (2.42–3.48)
LT (mm)	4.70 ± 0.78 (3.48–6.42)
VL (mm)	19.36 ± 1.59 (15.76–23.44)
STS (mm)	11.95 ± 0.79 (10.28–13.94)
ACV (mm^3^)	150.69 ± 30.58 (107–225)
Corneal astigmatism (D)	1.71 ± 0.55 (1.0–3.7)
Toric IOL axis (degree)	80.82 ± 51.13 (0–179)
Spherical power of IOL (D)	15.20 ± 3.40 (5.5–20)
K1(D)	42.51 ± 1.69 (37.8–46.7)
K2(D)	44.22 ± 1.61 (40.9–48.3)
Average keratometric value (D)	43.36 ± 1.63 (39.65–47.25)

*BCDVA* best corrected distance visual acuity, *logMAR* logarithms of the minimal angle of resolution, *AL* axial length, *ACD* anterior chamber depth, *LT* lens thickness, *VL* vitreous length; *STS* sulcus-to-sulcus, *ACV* anterior chamber volume, *D* diopter, *IOL* intraocular lens, *K1* flat meridian of keratometric value, *K2* steep meridian of keratometric value

**Table 2:** TECNIS toric intraocular lens model

Intraocular lens model	N (%)
ZCT100	4 (6.25)
ZCT150	27 (42.18)
ZCT225	20 (31.25)
ZCT300	8 (12.50)
ZCT400	5 (7.81)

### Postoperative visual acuity and refraction

**Fig. 1:**
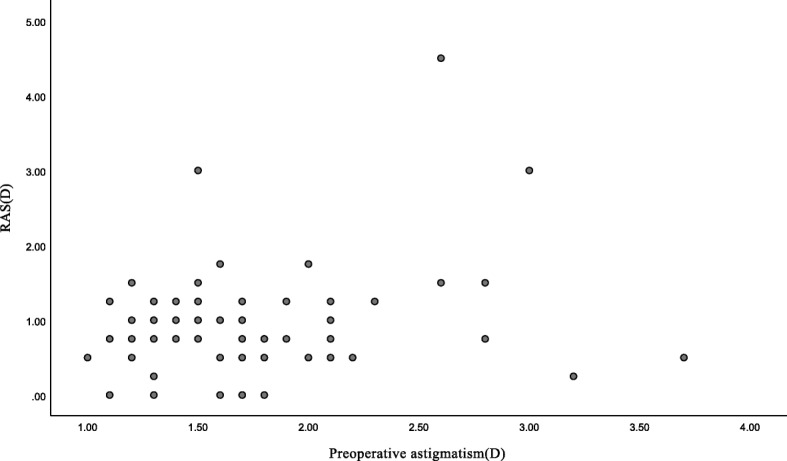
The distribution of the preoperative astigmatism and RAS. No significant correlation was observed between RAS and the preoperative astigmatism (Pearson’s *r* = 0.243, *p* = 0.053). *RAS* residual astigmatism

BCDVA was significantly improved from 0.93 ± 0.35 logMAR to 0.07 ± 0.10 logMAR after surgery at 3 months postoperatively. The RAS was 1.11 ± 0.75D and 0.91 ± 0.74 D at 1 month and 3 months after surgery, respectively. There was significant difference in RAS between 1 and 3 months (*p*<0.001). RAS was controlled within − 0.50 D in 34.37% (22/64) and − 1.00 D in 73.43% (47/64) of patients at 3 months. Table 3 shows RAS at 3 months after implantation of toric IOL. Figure 1 shows the distribution of the preoperative astigmatism and RAS. The proportion of patients with high astigmatism preoperatively among large RAS was 28.57% (2/7). Table 4 lists the distribution of preoperative astigmatism among large RAS.

**Table 3:** Residual astigmatism 3 months after toric IOL implantation

Residual astigmatism (D)	N (%)
≤ − 0.50	22 (34.37)
−0.51D to−1.00	25 (39.06)
-1.01D to-1.50	12 (18.75)
1.51D to −2.00	2 (3.12)
> − 2.00	3 (4.68)

*IOL* intraocular lens, *D* diopter

**Table 4:** RAS according to the preoperative astigmatism

Preoperative astigmatism (D)	RAS (D)
2.6	1.5
2.6	4.5
2.8	0.75
2.8	1.5
3	3
3.2	0.25
3.7	0.5

The proportion of patients with high astigmatism (>2.5D) preoperatively among large RAS was 28.57% (2/7). RAS residual astigmatism; D diopter

### State of toric IOL rotation

The mean toric IOL rotation was 7.42 ± 11.32° (0°-79°) and 7.48 ± 11.19° (0°-79°) at 1 and 3 months after surgery, respectively. The intraoperative mean area of capsulorhexis was 21.04 ± 3.30 mm^2^ for evaluating the correlation between the IOL rotation and the area of capsulorhexis. The mean area of capsulorhexis and the overlapped area between capsulorhexis and IOL optic was 21.00 ± 3.34 mm^2^ and 7.36 ± 2.82 mm^2^ at 1 month and 20.52 ± 3.31 mm^2^ and 7.34 ± 2.81 mm^2^ at 3 months postoperatively. The results indicated that 54.68% (35/64) patients were within 5° absolute rotation, and RAS within − 0.50 D and − 1.00 D was 34.37% (22/64) and 73.43% (47/64) at 3-month follow-up, respectively. Table 5 shows the IOL misalignment after toric IOL implantation.

**Table 5:** Intraocular lens misalignment after toric IOL implantation

IOL rotation (degrees)	3 Months N (%)	clockwise N (%)	Counter-clockwise N (%)
≤5	35 (54.68)	16 (25.00)	16 (25.00)
> 5 and ≤ 10	21 (32.81)	9 (14.06)	12 (18.75)
> 10 and ≤ 15	4 (6.25)	2 (3.12)	2 (3.12)
> 15	4 (6.25)	3 (4.68)	1 (1.56)

*IOL* intraocular lens

There were four eyes rotated more than 15°, and the greatest rotation reached 79°. In these four cases with significant rotation in our study, the AL ranged from 26.20 mm to 27.57 mm, and the corneal astigmatism ranged from 1.3 D to 3.0 D. The axis alignment of these cases was 158°, 88°, 92° and 81° intraoperatively and rotated 16°, 33°, 40° and 79° at 3 months after surgery, respectively. Table 6 lists the characteristics of four cases with large rotation.

**Table 6:** Characteristics of 4 large rotation cases (> 15°)

Parameters	Case1	Case2	Case3	Case4
ACD (mm)	2.67	2.63	2.71	2.77
ACV (mm3)	127	122	110	161
LT (mm)	4.4	6.15	4.34	4.51
VL (mm)	19.55	19.58	18.32	18.88
AL (mm)	27.34	26.78	26.2	26.96
Preoperative total corneal astigmatism (D)	1.3	3	2.6	1.5
Spherical power of IOL (D)	15	15	13	17
Toric IOL models	ZCT150	ZCT400	ZCT300	ZCT225
Intraoperative IOL axis (degree)	158	88	92	81
Area of capsulorhexis (mm2)	17.8	21.37	18.83	31.16
Area of overlap between capsulorhexis and optic (mm2)	10.46	6.89	9.43	1.58
K1(D)	44.4	43.4	43.2	40.4
K2(D)	45.7	46.4	45.8	41.9
Absolute value of rotation 1 month postoperatively (dgree)	16	33	40	79
Absolute value of rotation 3 months postoperatively (dgree)	16	33	40	79

In Case4, the area of capsulorhexis was largest (31.16 mm2), the area of overlap between capsulorhexis and optic was smallest (1.58 mm2). As a result, the toric IOL rotation reached 79°at 3 months after surgery

*ACD* anterior chamber depth, *ACV* anterior chamber volume, *LT* lens thickness, *VL* vitreous length, *AL* axial length, *D* diopter, *IOL* intraocular lens

### Factors associated with toric IOL rotation

**Fig. 2:**
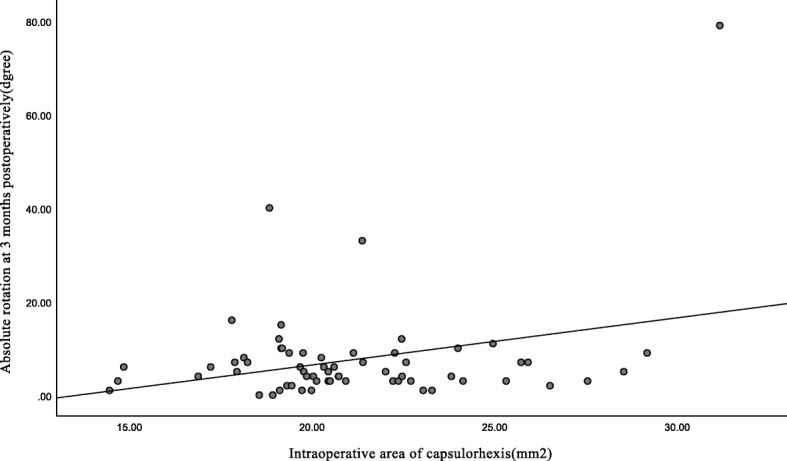
The correlation between Toric rotation and the area of capsulorhexis was assessed using Pearson’s correlation analysis. Toric IOL rotation was positively correlated with the area of capsulorhexis (Pearson’s r = 0.297, *p* = 0.017). *IOL* intraocular lens

The mean area of capsulorhexis was 21.04 ± 3.30 mm^2^, ranging from 14.45 to 31.16 mm^2^ intraoperatively. Toric IOL rotation was positively correlated with the intraoperative area of capsulorhexis (*y = 1.008x-13.724*, Pearson’s *r* = 0.297, *p* = 0.017) (Fig. 2). The variables included in the multiple linear regression analysis were age, gender, operated eye, preoperative BCDVA, preoperative total corneal astigmatism, AL, ACD, LT, VL, ACV, STS, keratometric value, spherical power of the implanted IOLs, intraoperative actual IOL axial direction, preoperative corneal astigmatism, the intraoperative area of capsulorhexis and the overlapped area between capsulorhexis and IOL optic. Table 7 shows the correlation analysis of toric IOL rotation as the dependent variable.

**Table 7:** Correlation between toric IOL rotation and the dependent variables

Variables	Pearson’s r	*P* Value
AL	−0.02	0.876
ACD	−0.113	0.387
LT	0.083	0.523
VL	− 0.082	0.546
STS	0.019	0.884
ACV	− 0.094	0.48
Keratometry K1	−0.101	0.429
Keratometry K2	−0.059	0.644
Intraopertive IOL axial direction	0.396	0.108
Preoperative corneal astigmatism	0.14	0.269
Area of capsulorhexis	0.297	0.017*
Area of overlap between capsulorhexis and optic	−0.191	0.131
Spherical power of IOLs	0.02	0.872

*Statistically significant correlation (*p*<0.05)

Toric IOL rotation was positively correlated with the area of capsulorhexis (Pearson’s *r* = 0.297, *p* = 0.017)

*IOL* intraocular lens, *AL* axial length, *ACD* anterior chamber depth, *LT* lens thickness, *VL* vitreous length, *STS* sulcus-to-sulcus, *ACV* anterior chamber volume, *K1* flat meridian of keratometric value, *K2* steep meridian of keratometric value

## Discussion

Astigmatism with long AL is the common refractive error of patients with cataract. Toshiyuki Miyake et al. have studied the distribution of corneal astigmatism in 12,428 eyes after cataract surgery and reported that 36.3% of eyes have more than 1.0 D of corneal astigmatism, 8.0% have more than 2.0 D, and 2.4% have more than 3.0 D [10]. Postoperative misalignment is a significant problem in toric IOL implantation. Therefore, we observed the clinical outcomes and evaluated rotational stability and its possible related factors, including preoperative biometric examinations and surgical operation.

In our retrospective study, the mean RAS and absolute IOL rotation were 0.91 ± 0.74 D, ranging from 0 to-4.5D, and 7.48 ± 11.19° (0–79°) at 3 months after surgery, respectively, which were larger than those in the eyes with various axial length in other studies [5, 11]. This might be attributed to the fact that all patients had long AL or a higher percentage of high myopia. No significant differences were observed in toric IOL rotation between 1 and 3 months, suggesting that toric IOL rotation mainly occurred within 1 month after surgery [12]. Compared with toric IOL implantation in eyes of emmetropia, hyperopia or AL shorter than 25 mm of other studies [13–16], the higher rate of IOL rotation would occur in patients with long AL probably.

We established that Toric IOL rotation was positively correlated with the area of capsulorhexis (*y* = *1.008x-13.724,* Pearson’s *r* = 0.297, *p* = 0.017), while there was no correlation with the overlapped area between capsulorhexis and IOL optic. These findings suggested that an appropriately sized CCC was essential to prevent IOL rotation, especially at early stage after surgery. A relatively smaller sized CCC was conducive than oversize one. There were significant negative correlations between the area of CCC and the overlapped area between capsulorhexis and IOL optic (*p* < 0.001, *r* = − 0.979). Moreover, we didn’t find a correlation between IOL rotation and AL, LT, VL, ACD, STS, keratometry value, the spherical power of the implanted IOL.

**Fig. 3:**
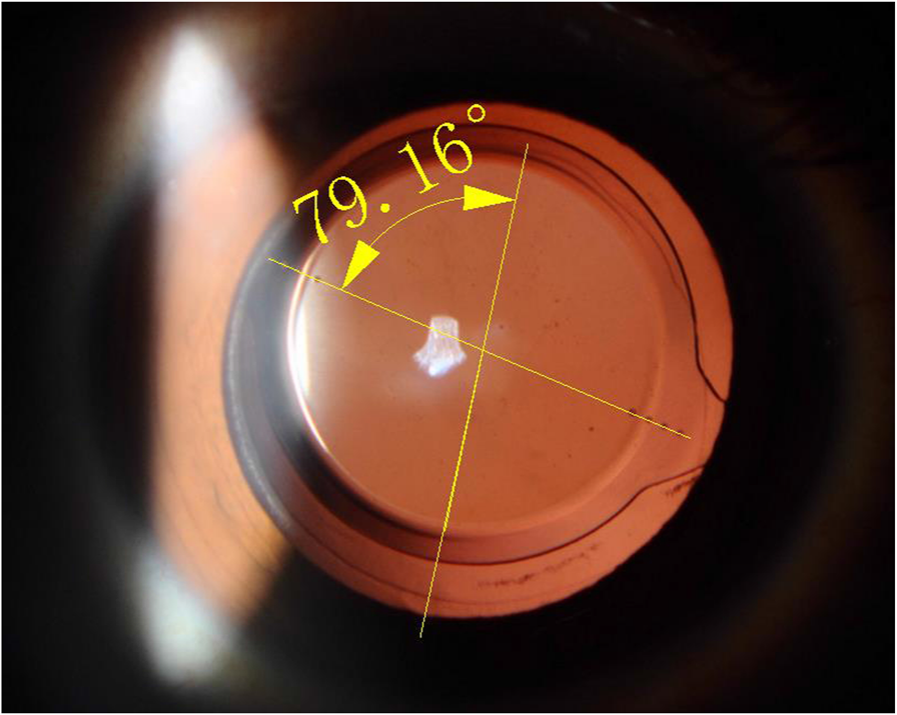
Photograph showed the rotation of Case 4 by Rhinoceros 5.0. The edge of IOL was almost free and it rotated 79° at 3 months after surgery. *IOL* intraocular lens

Four cases in our study rotated more than 15°. There were no significant differences between the four cases and other cases in terms of average ACD, ACV, LT, AL, the intraoperative area of CCC, and the area of overlap between capsulorhexis and IOL optic. Among the data, the case in which toric IOL rotation reached 79° at 3 months after surgery had a significantly larger intraoperative area of capsulorhexis than the average of others. The capsulorhexis size was too large to fix the toric IOL, and the edge of IOL was almost free, therefore leading to IOL rotated extensively (Fig. 3). It was observed in the two cases which rotated 33° and 40° without too large capsulorhexis size, including larger preoperative corneal astigmatism, intraoperative approximately vertical toric IOL alignment. No correlations were found between IOL rotation and the variables, including keratometric value, intraoperative IOL axial direction, preoperative corneal astigmatism. Therefore, accumulation of more cases and a long-term observation should be taken in future.

During the first 3 months after surgery, several studies have mentioned that the toric IOL rotation can be attributed to an IOL surface with a low coefficient of friction [17], thinner optic of low-powered IOLs [10], the design of the IOL [18], an overall length of the IOL that is too small for the capsular bag [19] and instability of the anterior chamber [20].

Some earlier studies have found a positive correlation between early rotation of the toric IOL and AL, which is positively correlated with the diameter of the capsular bag [21–23]. Since the diameter of capsular bag cannot be measured directly, we measured STS using Anterior Segment Visante™ OCT, which is a non-contact optical signal acquisition. We didn’t find a correlation between AL and STS (Pearson’s *r* = − 0.005, *p* = 0.971), suggesting that AL was not positively correlated with the diameter of the capsular bag in a long AL eye.

Other earlier literature has also indicated that the area of CCC was contractible at 3 months compared with 1 month after surgery. When the collapses and fibroses of capsular bag occur, the area of CCC would decrease and influenced the IOL rotation stability [24]. In our observation, compared with intraoperative one, the area of CCC was decreased slightly at 1 month postoperatively and continued to be decreased at 3 months postoperatively. Meanwhile, the axis of implanted toric IOL was not changed in the 3-month follow-up.

There are some limitations in our study. The digital anterior segment photographs were used to calculate the area of capsulorhexis as well as the overlapped area between capsulorhexis and IOL optic. This process was related with the subjectivity of the operator. In order to reduce the influence of inaccurate calculation, we repeated the measurement for three times. The toric IOL alignment axis marker was at 10° intervals, and manual marking was used. VERION system has the advantage of intraoperative digital guidance of the toric IOL alignment even for 1° [25]. It has been reported that the underlying capsular shrinking probably occurs at 6 months or 1 year after cataract surgery, with the beginning in the first 3 months [26]. In order to reduce the postoperative RAS seized with SIA, we evaluated the early-stage outcomes until 3 months after the surgery in this study. Inevitably, some cases might already suffer from a very early-stage fibrosis and contraction of the capsular bag at this time.

Our data showed the refractive results and the factors influencing toric IOL rotation in eyes with long AL. The area of CCC affected their early-stage stability of toric IOL axis after surgery. However, the long-term influence from the capsular bag contraction and posterior capsular opacity should be considered.

## Conclusions

Over all, the large CCC was a risk factor for toric IOL rotation in eyes with long AL. An appropriately smaller sized CCC was conducive to increase the rotational stability rather than an oversize one.

## Supplementary information

**Additional file 1.** Data for statistic analysis.

## Data Availability

The datasets used and/or analyzed during the current study are available from the corresponding author on reasonable request.

## Abbreviations

IOL: Intraocular lens
AL: Axial length
D: Diopter
ACD: Anterior chamber depth
LT: Lens thickness
VL: Vitreous length
ACV: Anterior chamber volume
STS: Sulcus-to-sulcus
K: Keratometric value;
BCDVA: Best corrected distance visual acuity
logMAR: Logarithms of the minimal angle of resolution
RAS: Residual astigmatism degree
LASIK: Laser-assisted in situ keratomileusis
TCA: Total corneal astigmatism;
QS: Quality safety
UCDVA: Uncorrected distance visual acuity
OCT: Optical coherence tomography
SIA: Surgically induced astigmatism
CCC: Continuous curvilinear capsulorhexis
SD: Standard deviation
